# The Pay of the Nurse.—IV. The Ill-Effects of the Late Start in Life
*Previous articles appeared on March 5, 12, and 26.


**Published:** 1921-04-02

**Authors:** 


### THE MATRONS' AND SISTERS' DEPARTMENT.

### THE PAY OF THE NURSE.*

### IV.—The Ill-effects of the Late Start in Life.

Few nurses complete their training before the age of twenty-five. Many do not do so till they are twenty-six or older. For after three or four years of training in a general hospital there are special qualifications to be added on, fever, midwifery, a public health course, district training, etc., any of which may take from six to nine months, according to how the necessary course can be fitted in. At twenty-six, then, the nurse begins to practise her profession. Meantime what have her friends and relatives been doing in other careers?

We are at once confronted with the fact that the nursing profession is adopted (1) by women who usually commence work on their own account about the age of sixteen, and (2) by women who look to continue their studies till they are eighteen, or in the case of university students, about twenty-two. In both cases they are adversely affected by the conditions prevailing in the nursing profession at the present time. Whether the woman begins work early or remains in the shelter of home or collegiate life till her faculties have been more fully developed, it is unreasonable and contrary to the practice followed in other callings to treat her as a novice, to whom a bare minimum wage is due, at the age of twenty-six.

Women who begin the battle of life at the age of sixteen, whether formally admitted as apprentices or not, expect to spend some years at any rate in the lower ranks of employment. They may be clerks, elementary school-teachers, members of the staff in business and commercial houses, telegraphists, to name but a few callings, but one and all within ten years they will be well on their way to a secure position and a good proportion will have already attained it before thirty. These are the marrying years, and though we are speaking of the women who do not marry early, the marriage factor is a very important one. The high percentage of marriage among working women between the ages of twenty and thirty lessens competition and brings more opportunity to women of ability who do not marry. Out of every hundred women who start their career together only a smallish percentage continue at it as long as ten years, and naturally there is more chance under this weeding-out process for the remainder. Accordingly, while the nurse fresh from her training-school, after waiting five years to begin and spending four or five years over her apprenticeship, is still a novice and *paid at novice rates*, her sister is head of a department in her office, or is in charge of a branch, or has obtained a headmistressship. She has had time to prove her worth, and is valued accordingly. The only possible way to compensate the nurse for her long preliminary sacrifice of time is to pay her the salary to which a woman of twenty-six, thoroughly well equipped as regards professional knowledge and skill, is entitled. Only by this course can the supply of women able and willing to make the necessary sacrifice of ten earning years be kept up.

There are a few professions besides nursing in which the entrant cannot be fairly launched before the age of twenty-six. Such are the medical professions, the higher branches of the Civil Service, inspectorships, and the like. But no one could afford to enter for these posts unless the commencing salary were adequate to cover the long preparation, not to speak of the promise of valuable advancements. We are not going to induce university women in any numbers to enter the nursing profession, so long as the rewards of their perseverance remain as nebulous as they are at present. It is quite true that the nurse's training, though long and arduous, is not costly. But it requires just that surrender of the life which women find it increasingly difficult to make. There is no fear that it would not gladly be embraced by hundreds of highly educated women who regard the nurse's career with admiration, if at the end of the long road there were a prospect of reasonable independence and an income commensurate with that obtainable in other directions. What should we think of the man's case who, after spending four or five of his best earning years in acquiring proficiency, should discover his earning capacity to be from £55 to £80 a year, coupled with the condition of continuing to live under a system of tutelage?

Considerations of this nature must convince the members of the profession most disposed to be satisfied with things as they are that there is something to be said for the very modest scale of reformed salaries put forth by the College of Nursing. More and more the profession needs women who have the necessary mental training to teach and organise. It wants them for important posts in public health work. It wants them to direct properly co-ordinated centres of district work, where the lack of organisers is keenly felt. It wants them to take the direction of private nursing and develop new methods applicable to changed social conditions. It wants them for the training of backward races, for a thousand purposes of the highest international importance. But it will not get them until the pitiful pay often given with a tacit understanding that the nurse's devotion must serve her in place of salary is replaced by just remuneration.

* Previous articles appeared on March 5, 12, and 26.

